# IFN-γ-Based ELISpot as a New Tool to Detect Human Infections with Borna Disease Virus 1 (BoDV-1): A Pilot Study

**DOI:** 10.3390/v15010194

**Published:** 2023-01-10

**Authors:** Lisa Eidenschink, Gertrud Knoll, Dennis Tappe, Robert Offner, Thomas Drasch, Yvonne Ehrl, Bernhard Banas, Miriam C Banas, Hans Helmut Niller, André Gessner, Josef Köstler, Benedikt M J Lampl, Matthias Pregler, Melanie Völkl, Jürgen Kunkel, Bernhard Neumann, Klemens Angstwurm, Barbara Schmidt, Markus Bauswein

**Affiliations:** 1 Institute of Clinical Microbiology and Hygiene, University Hospital Regensburg, 93053 Regensburg, Germany; 2 Bernhard Nocht Institute for Tropical Medicine, 20359 Hamburg, Germany; 3 Department of Transfusion Medicine, Institute of Clinical Chemistry and Laboratory Medicine, University Hospital Regensburg, 93053 Regensburg, Germany; 4 Department of Nephrology, University Hospital Regensburg, 93053 Regensburg, Germany; 5 Institute of Medical Microbiology and Hygiene, University of Regensburg, 93053 Regensburg, Germany; 6 Regensburg Department of Public Health, 93059 Regensburg, Germany; 7 Department of Epidemiology and Preventive Medicine, University of Regensburg, 93053 Regensburg, Germany; 8 Department of Pediatrics, University Hospital Regensburg, 93053 Regensburg, Germany; 9 Department of Neurology, Donau-Isar-Klinikum Deggendorf, 94469 Deggendorf, Germany; 10 Department of Neurology, University of Regensburg, Bezirksklinikum, 93053 Regensburg, Germany

**Keywords:** Borna disease virus 1 (BoDV-1), zoonosis, encephalitis, diagnostics, immunopathology, ELISpot, T cells, IFN-γ, peptides

## Abstract

More than 40 human infections with the zoonotic Borna disease virus 1 (BoDV-1) have been reported to German health authorities from endemic regions in southern and eastern Germany. Diagnosis of a confirmed case is based on the detection of BoDV-1 RNA or BoDV-1 antigen. In parallel, serological assays such as ELISA, immunoblots, and indirect immunofluorescence are in use to detect the seroconversion of Borna virus-reactive IgG in serum or cerebrospinal fluid (CSF). As immunopathogenesis in BoDV-1 encephalitis appears to be driven by T cells, we addressed the question of whether an IFN-γ-based ELISpot may further corroborate the diagnosis. For three of seven BoDV-1-infected patients, peripheral blood mononuclear cells (PBMC) with sufficient quantity and viability were retrieved. For all three patients, counts in the range from 12 to 20 spot forming units (SFU) per 250,000 cells were detected upon the stimulation of PBMC with a peptide pool covering the nucleocapsid protein of BoDV-1. Additionally, individual patients had elevated SFU upon stimulation with a peptide pool covering X or phosphoprotein. Healthy blood donors (*n* = 30) and transplant recipients (*n* = 27) were used as a control and validation cohort, respectively. In this pilot study, the BoDV-1 ELISpot detected cellular immune responses in human patients with BoDV-1 infection. Its role as a helpful diagnostic tool needs further investigation in patients with BoDV-1 encephalitis.

## 1. Introduction

Infections with Borna disease virus 1 (BoDV-1) cause severe courses of encephalitis in humans [1,2,3]. Since the confirmation of the first zoonotic cases in 2018, more than 40 infections within the endemic regions of southern and eastern Germany have been reported to German health authorities [3,4,5,6,7,8]. The bicolored white-toothed shrew (*Crocidura leucodon*) has been described as at least one reservoir [9,10]. The exact transmission route as well as the incubation period are unknown. After first unspecific prodromal symptoms, infections lead to severe neurological disease with dizziness, memory loss, ataxia, seizures, apnea, and deep coma. Some cases resemble a Guillain-Barré or Miller-Fisher syndrome [4]. The case fatality rate is extremely high (>90%) [11]. For the confirmation of a BoDV-1 infection, the detection of BoDV-1-specific RNA by RT-qPCR/in situ hybridization or the detection of BoDV-1 protein by immunohistochemistry from cerebrospinal fluid (CSF), brain biopsies or tissue samples is required [5]. Additionally, serological assays such as indirect immunofluorescence (iIFA), ELISA, and immunoblots have been established [1,5,7]. Since only a comparatively low amount of BoDV-1 RNA seems to be shed into the CSF, the sensitivity of RT-qPCR from this material is limited, while it reliably detects BoDV-1 RNA in brain biopsies or brain tissue samples [3]. A positive serology without the detection of BoDV-1 RNA in the CSF is classified as a probable case [5]. In rare cases, an invasive brain biopsy or post-mortem tissue samples are required for the definitive confirmation of the diagnosis. In most cases, IgG seroconversion does not occur before severe neurological symptoms are present [7]. The pathogenesis of Borna disease has mainly been attributed to T cell-based immune responses [12,13]. T cells are present in virus-infected brain regions of BoDV-1 patients, with CD4 positive cells outnumbering CD8 positive cells [12]. In addition, an elevation of pro-inflammatory markers such as IFN-γ, IL-6, and chemokines (CCL-2, CCL-5, CXCL-10, IL-8) has been described in the serum and CSF of patients suffering from BoDV-1 infection [14]. As for tuberculosis and cytomegalovirus infections, the detection of pathogen-specific T cells is already exploited for diagnostic tests [15], we addressed the question of whether BoDV-1 infections can be diagnosed using a modified IFN-γ-based ELISpot with overlapping peptide pools of three BoDV-1-specific proteins.

## 2. Materials and Methods

### 2.1. Patients and Samples

The diagnosis of a BoDV-1 infection was confirmed by RT-qPCR from CSF and/or post-mortem neuronal tissue for all patients. Patient 1 was the patient published by Neumann et al. [7]. Patient 2 was a thus far unpublished 10–20-year-old patient who was diagnosed at the Institute of Clinical Microbiology and Hygiene, University Hospital Regensburg, Regensburg, Germany. Patient 3 was a thus far unpublished 50–60-year-old patient diagnosed at the same institute. The exact age and gender cannot be revealed due to the ethical requirements. Test results confirming the BoDV-1 infection are shown in Table 1. Patients 1–3 were diagnosed between 2020 and 2022. Peripheral blood mononuclear cell (PBMC) samples of four additional patients with confirmed BoDV-1 infection were kindly provided by the Bernhard Nocht Institute for Tropical Medicine, Hamburg, Germany. The retrospective examination of clinical samples of patients with encephalitis for the detection of new viruses such as BoDV-1 was approved by the Ethics Committee of the Faculty for Medicine, University of Regensburg, Regensburg, Germany (reference number: 18-1248-101).

As the respective control and validation samples, PBMC samples from healthy blood donors (*n* = 30) and outpatients after solid-organ transplantation (*n* = 27) were included, which were obtained as part of the BoSOT study (BoSOT: “BoDV-1 after solid-organ transplantation”). The BoSOT study was conducted in an endemic region of southern Germany with recently confirmed BoDV-1 infections in humans and animals [3,7]. The study was approved by the Ethics Committee of the Faculty for Medicine, University of Regensburg, Regensburg, Germany (reference number: 21-2202-101) and registered in the DRKS trial register (DRKS00025180) on 4 May 2021. As participants, 216 healthy blood donors at the Department of Transfusion Medicine, Institute of Clinical Chemistry and Laboratory Medicine, University Hospital Regensburg, Regensburg, Germany were included in the study from 2 November 2021 until 18 May 2022. The Department of Nephrology, University Hospital Regensburg, Regensburg, Germany, enrolled 280 outpatients at routine follow-up after solid-organ transplantation from 21 May 2021 until 12 January 2022. All study participants of the BoSOT study (all aged ≥18 years) were included after informed consent. Blood samples for research purposes were drawn as part of a routine venipuncture, either for the purpose of a blood donation (blood donors) or for the routine check-up (transplant patients). No additional venipuncture for research purposes only was performed. The serum of all participants was screened for anti-BoDV-1-antibodies using an in-house ELISA [7], followed by an iIFA as the confirmatory test. Lithium heparin blood for ELISpot diagnostics was drawn in parallel from sub-cohorts.

### 2.2. BoDV-1 RT-qPCR

RNA extraction and BoDV-1 specific RT-qPCR were performed as described recently [1,7]. Mixes 1 and 6 were used for the detection of BoDV-1 RNA within the *X/P* and *M/G* genes, respectively. In vitro-transcribed RNA molecules were used as standards for quantification.

### 2.3. BoDV-1 iIFA

An iIFA was performed following a recently published protocol [1]. Vero cells persistently infected with BoDV-1 were mixed 1:2 with uninfected Vero cells and cultured overnight in 96-well microtiter plates (Ibidi, Gräfelfing, Germany) to achieve confluent cell layers. Wells with uninfected Vero cells served as the negative control. After removal of the supernatant, plates were dried for 2 h and then fixed at 80 °C for 2 h. Thereafter, heat-inactivated serum/plasma samples were added in a 2-fold dilution series in TRIS buffer (Sigma-Aldrich, St. Louis, MO, USA) (1:20, 1:40, 1:80). After incubation for 1 h, plates were washed three times with DPBS and incubated with a 1:200 dilution of Cy-3-conjugated polyclonal rabbit anti-human-IgG antibody (Jackson ImmunoResearch, West Grove, PA, USA) for 1 h. After a final washing step, the assays were analyzed using fluorescence microscopy. Samples with characteristic fluorescing spots in the nuclei of the BoDV-1-infected Vero cells were considered positive. When similar fluorescence signals were detected in non-infected and BoDV-1-infected Vero cells, samples were considered unspecific. All samples were evaluated separately by two trained and experienced members of the laboratory staff.

### 2.4. Isolation of PBMC

PBMC were isolated from lithium heparin blood by density gradient centrifugation. Lithium heparin blood was diluted 1:1.6 by pre-warmed RPMI 1640 medium (PAN-Biotech, Aidenbach, Germany) before it was transferred to a LeucoSEP tube (Greiner Bio-One, Frickenhausen, Germany). Density gradient centrifugation was performed at 1000 rcf without acceleration and break for 10 min at room temperature (RT). The PBMC fraction was obtained and washed in RPMI medium twice before the cell suspension was adjusted to 2,500,000 viable cells/mL in AIM-V medium with the AlbuMAX supplement (Thermo Fisher Scientific, Waltham, MA, USA). Viable cells were counted by an automated cell counter according to the manufacturer’s recommendations (Vi-CELL XR, Beckman Coulter, Brea, CA, USA).

### 2.5. Storage of PBMC

If the PBMC samples could not be analyzed immediately, they were frozen in 7.5% DMSO (15% DMSO (AppliChem, Karlsruhe, Germany) in FCS (PAN-Biotech, Aidenbach, Germany), diluted 1:2 with AIM-V medium) within cryotubes and stored at −80 °C.

### 2.6. BoDV-1 ELISpot

Wells pre-coated with anti-human-IFN-γ (Human IFN-γ ELISpot Pro Kit (ALP), Mabtech, Nacka Strand, Sweden) were washed four times with 200 µL sterile DPBS (Gibco, Thermo Fisher Scientific, Waltham, MA, USA) and then equilibrated with 200 µL AIM-V medium (30 min at RT) according to the manufacturer’s instructions. The AIM-V medium was removed and then 250,000 cells (in 100 µL AIM-V) were seeded per well. As negative control, 50 µL of the AIM-V medium only were added to the cells. For stimulation of virus-specific T cells, BoDV-1-derived 11aa-overlapping 15-mer peptide pools spanning full-length viral nucleoprotein (N), accessory X protein, and phosphoprotein (P) were used (peptides and elephants, Hennigsdorf, Germany) (Table S1). Peptides were dissolved in H_2_O, adjusting the concentration of stock solutions to 200 µg/mL per single peptide. For stimulation of PBMC, a final concentration of 1 µg of each single peptide in 150 µL final volume per well was adjusted in AIM-V medium. A total of 50 µL of phytohemagglutinin (PHA) solution (T-SPOT.TB Kit, Oxford Immunotec, Abingdon, UK) served as stimulation for the positive control. All plates were incubated at 37 °C and 5% CO_2_ for 17–20 h. After four washing steps with 200 µL DPBS, wells were stained with 50 µL anti-human-IFNγ-AP (T-SPOT.TB Kit) (1:200 dilution in DPBS) at 4 °C for 1 h. After four washing steps with 200 µL DPBS, spots were developed with NBT/BCIP substrate solution (T-SPOT.TB Kit) at RT for 7 min. Reaction was stopped with H_2_O. Plates were dried at 37 °C for 1.5–2 h. Spot forming units (SFU) were analyzed by an AID iSpot Robot (AID Autoimmun Diagnostika, Straßberg, Germany) with the following settings: relative spot intensity ≥ 25 (intensity in brightness units relatively to the background), spot size ≥ 100 [0.01 mm^2^], gradient ≥ 10. The test was considered invalid if there were more than 10 SFU/250,000 PBMC in the negative control or less than 20 SFU/250,000 PBMC in the positive control. The SFU count of the matched negative control was subtracted from the corresponding wells stimulated with BoDV-1 peptides or PHA for further analysis. A flowchart for the ELISpot procedure is provided in Figure S1.

### 2.7. Statistical Analysis

Graphs and statistical analysis were created using GraphPad Prism, version 9.4.1 (GraphPad Software, San Diego, CA, USA). For statistical analysis, the non-parametric Kruskal–Wallis test, followed by Dunn’s multiple comparisons test, were performed (ns *p* > 0.05; * *p* ≤ 0.05; ** *p* ≤ 0.01; *** *p* ≤ 0.001; **** *p* ≤ 0.0001).

## 3. Results

PBMC samples of three patients with a proven BoDV-1 infection that had been diagnosed at our institute between 2020 and 2022 were available for ELISpot diagnostics. Diagnostic tests confirming BoDV-1 infection are shown in Table 1. In addition, we received frozen PBMC samples of four further patients with BoDV-1 infection from another center (time interval between freezing and ELISpot testing in the range of 0.5 to 3 years). As controls, PBMC samples from healthy blood donors (*n* = 30) with negative BoDV-1 serology (based on iIFA) were utilized. PBMC samples of outpatients after solid-organ transplantation (*n* = 27) with a negative BoDV-1 serology (based on iIFA) served as a BoDV-1-negative validation cohort.

PBMC samples of the control and validation cohort as well as of one patient with BoDV-1 infection (patient 3) were directly analyzed with the ELISpot test after preparation; all other PBMC samples of patients with BoDV-1 infection had been frozen prior to testing (time interval between freezing and thawing was 12 months for patient 1 and 17 months for patient 2).

For three of four PBMC samples that were provided to us by an external center, counts of viable cells were not sufficient to follow the established protocol. Considering the restricted availability of PBMC samples of patients suffering from BoDV-1 infection, the ELISpot was carried out with lower cell concentrations (see Table S2), but these results were excluded from further analysis.

For the BoDV-1 ELISpot, PBMC samples were stimulated with a peptide pool either covering the full-length viral nucleocapsid (N), X or phosphoprotein (P) by 15-mer peptides with an overlap of 11 amino acids (Table S1).

Samples with a spot forming unit (SFU) count lower than 20 SFU/250,000 PBMC in the PHA control were excluded from further analysis (the fourth sample of a patient with BoDV-1 infection that was provided by the external center, see Table S2).

**Figure 1:**
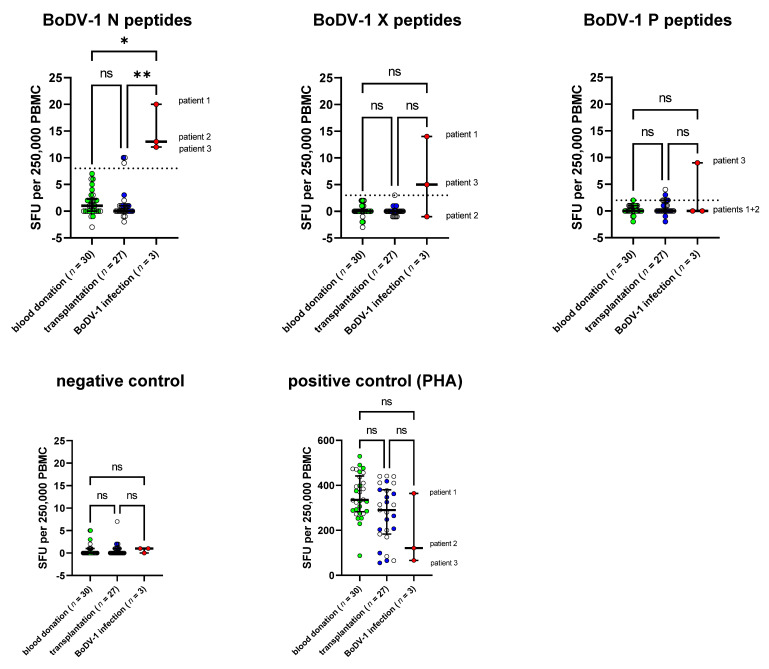
Spot counts of BoDV-1 ELISpot. PBMC samples of three patients with BoDV-1 infection were of sufficient viability to be stimulated with peptide pools comprising either the BoDV-1 N, X or P protein (15-mer peptides with an overlap of 11aa). Patient 2 and patient 3 were under immunosuppressive treatment at the time of sampling. PBMC cultivated in medium only served as the negative control, PBMC stimulated with phytohemagglutinin (PHA) as the positive control. Counts of matched negative controls were subtracted from other conditions for further analysis. For the negative control, the uncorrected counts are shown. In comparison, healthy blood donors (*n* = 30) and patients after solid-organ transplantation (*n* = 27) were tested. Spot forming units (SFU) are shown for the seeded 250,000 PBMC. Lines represent the median with interquartile range (IQR). SFU counts of patients with BoDV-1 infection were significantly elevated for stimulation with the N peptide pool based on a Kruskal-Wallis test followed by Dunn’s multiple comparisons test (ns *p* > 0.05; * *p* ≤ 0.05; ** *p* ≤ 0.01). Dotted horizontal lines represent the calculated cut-offs for stimulation with each peptide pool, based on the data of blood donation (mean + 3 standard deviations).

For the remaining three samples of patients with confirmed BoDV-1 infection, the SFU/250,000 PBMC was 20, 13, and 12 upon stimulation with the BoDV-1 N peptide pool, while the maximum SFU/250,000 PBMC in the control cohort of healthy blood donors was 7 (Figure 1 and Figure S2). In addition, SFU counts upon stimulation with the BoDV-1 X peptide pool were 14 SFU/250,000 PBMC for patient 1 and 5 SFU/250,000 PBMC for patient 3, while maximum counts for the healthy blood donors were 2 SFU/250,000 PBMC. When PBMC was stimulated with BoDV-1 P peptides, patient 3 had 9 SFU/250,000 PBMC, while the maximum count was 2 SFU/250,000 PBMC for the control cohort of healthy blood donors. For stimulation with the N peptide pool, the group of patients with BoDV-1 infection was significantly different from both the control group of healthy blood donors as well as from the cohort of patients after solid-organ transplantation based on a Kruskal-Wallis test, followed by Dunn’s multiple comparisons test as a post hoc test, while the effects were not significant for stimulation with X or P peptides. Of note, patient 2 and patient 3 received immunosuppressive medication at the time of sampling.

To standardize the test interpretation, cut-offs for each stimulation condition were calculated as the mean plus three standard deviations of the control cohort of healthy blood donors. This resulted in a cut-off of >8 SFU for stimulation with the N peptide pool, >3 SFU for stimulation with the X peptide pool, and >2 SFU for stimulation with the P peptide pool. Based on these cut-offs, none of the test samples from the healthy blood donor cohort was positive.

Test sensitivity based on the calculated cut-off was 100% for stimulation with the N peptide pool, 67% for stimulation with the X peptide pool and 33% for stimulation with the P peptide pool, whereby one patient with BoDV-1 infection (33%) showed reactivity upon all three stimulation conditions and two patients (67%) upon at least two stimulation conditions.

**Figure 2:**
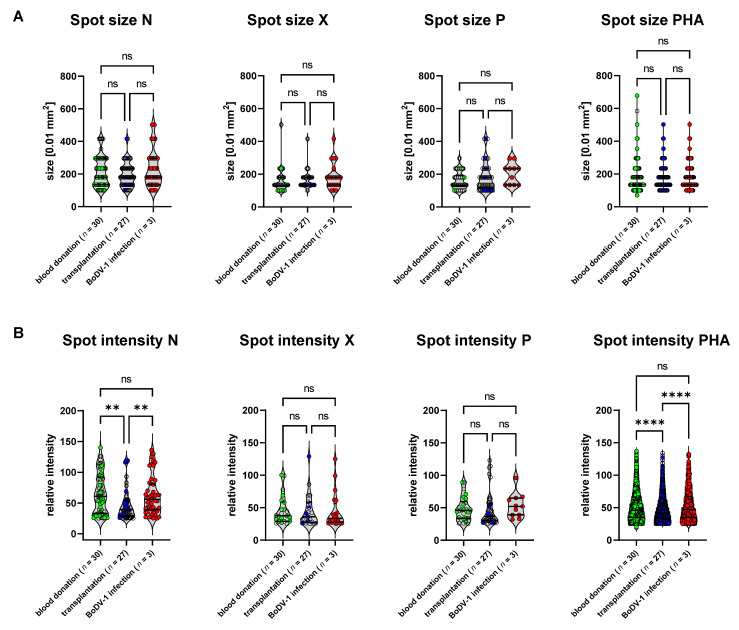
Spot sizes and spot intensities of BoDV-1 ELISpot. Distributions of spot sizes [0.01 mm^2^] (**A**) and spot intensities (**B**) are depicted as violine plots (horizontal lines: median and quartiles) for different conditions (stimulation with BoDV-1-protein-derived peptides or PHA) and cohorts. For statistical analysis, a Kruskal-Wallis test followed by Dunn’s multiple comparisons test was performed (ns *p* > 0.05; ** *p* ≤ 0.01; **** *p* ≤ 0.0001).

For the validation of the specificity of the ELISpot and its cut-offs, a cohort of outpatients after solid-organ transplantation (*n* = 27) with negative BoDV-1 serology was included. This cohort represented patients 1 to 23 years after kidney or combined kidney/pancreas transplantation. All patients were treated with a similar immunosuppressive protocol according to the local standard of the transplantation center (see Table S3). The rationale for including this validation group was the assumption that this cohort represents a target group for BoDV-1 tests when differential diagnoses of post-transplantation encephalopathy/encephalitis need to be assessed, as at least five cases of human BoDV-1 infections have been described in patients with a history of solid-organ transplantation [1,3]. On the other hand, the complex immunological issues of this cohort challenge immune-based tests such as our previously described BoDV-1 ELISA, for which test specificity was significantly reduced in this cohort (data not shown). The complex and variable immune history within this group caused by different underlying diseases, time since transplantation, organ rejection, AB0-incompatibility, and differences in immunosuppressive medication might be reflected by the variance of SFU counts upon PHA stimulation in our BoDV-1 ELISpot (Figure 2). In addition, there were differences in spot intensities upon stimulation with BoDV-1 N peptides as well as PHA for this cohort (Figure 2B), while the spot sizes and spot intensities did not differ between the control group of healthy blood donors and patients suffering from BoDV-1 infection (Figure 2A,B). Test specificity of the BoDV-1 ELISpot was calculated for this subgroup of patients after solid-organ transplantation and resulted in a specificity of 89% for stimulation with the N peptides, 100% for stimulation with the X peptides, and 93% for stimulation with the P peptides, whereby one patient after solid-organ transplantation was tested to be reactive in two stimulation conditions (N and P).

Thus, for the overall test interpretation, we suggest a positive result upon stimulation with N peptides as the most important criterion (sensitivity 100%, specificity 89%). An additional criterion of a second positive result upon stimulation with X or P peptides increased the specificity to 96%, but decreased the sensitivity to 67%.

Comparing the ELISpot results of the patients with BoDV-1 infections to a BoDV-1 ELISA based on recombinant proteins [7], positive ELISpot results were only partially paralleled by positive ELISA results against the corresponding antigen (Table 2).

An elevated SFU in BoDV-1 ELISpot upon stimulation with the X-derived peptides was paralleled by a positive anti-BoDV-1-X-IgG ELISA in patient 1. Similar results were found for the N protein in patient 2 and the P protein in patient 3, while positive ELISpot results for the N protein were not paralleled by a positive anti-BoDV-1-N-IgG ELISA in patients 1 and 3.

## 4. Discussion

ELISpot diagnostics were first introduced for tuberculosis [15]. Subsequently, the test was adapted for the detection of CMV- and SARS-CoV-2-specific T-cell responses [16,17,18,19,20].

The immunopathology of BoDV-1 infections has mainly been attributed to T-cell responses [13]. IgG seroconversion occurs late during the course of the disease when severe neurological symptoms are already present [7]. Given the T-cell pathology of Borna disease and the previously described elevation of IFN-γ in the serum and CSF of patients with this infection [14], we addressed the question of whether an IFN-γ-based ELISpot is suitable in diagnosing patients with BoDV-1 infections. As we previously measured the serological responses against BoDV-1 N, X, and P protein by an ELISA system, we utilized 15-mer peptides covering these proteins for the stimulation of isolated PBMC. By choosing peptide pools spanning the full-length proteins, we ensured the test performed independently from the patients’ HLA alleles.

All three patients with confirmed BoDV-1 infection from whom a sufficient number of viable PBMC was retrieved and whose positive control fulfilled the inclusion criteria had elevated SFU counts upon stimulation with the N peptide pool. In addition, the PBMC of two patients led to elevated spot numbers upon stimulation with the X and the P peptide pool, respectively.

Our data show for the first time that BoDV-1 N protein is a major target of cellular immunity in human BoDV-1 infections. In line, a peptide of the N protein was previously identified as an epitope of CD8 positive T-cell responses in an infection model of Lewis rats [21,22]. Another study in mice identified the N-peptide TELEISSI as the immunodominant T-cell epitope [23]. In a study with rats, an early N-specific CD8 positive T cell response was found to prevent immune pathology in the brain [24]. As we measured the total PBMC, no conclusions can be made as to whether the IFN-γ signal is derived from CD4 or CD8 positive T cells in our test.

In two cases (patients 1 and 3), a positive BoDV-1 ELISpot SFU count upon stimulation with N-derived peptides was not paralleled by a positive anti-N-IgG ELISA result based on recombinant N protein. Using membrane-based linear epitope mapping with 15-mer N-derived peptides spanning the whole BoDV-1 protein with 11 amino acids overlapping, IgG reactions against two N-derived peptides were found for patient 1, but not for patient 3 (data not shown). Therefore, at least in some cases, a peptide-based serological approach, as it has also been applied in early serological studies [25], might compare more favorably to a peptide-based BoDV-1 ELISpot.

While in the acute phase of BoDV-1 infections, experimental animal data found non-neutralizing antibodies against the BoDV-1 N and P protein, neutralizing antibodies against the BoDV-1 M and G protein were detected in the chronic phase [26]. For monoclonal anti-BoDV-1-G-protein antibodies a prophylactic effect in preventing encephalitis has been demonstrated [26]. In further studies, cellular immune responses against BoDV-1 M and G protein that were not addressed by our current BoDV-1 ELISpot approach might also be of interest.

The ELISpot technology with the preparation of PBMC was developed in order to improve the test performances for patients under immunosuppression. The relative robustness of the tuberculosis ELISpot under the condition of immunosuppression, for example, in patients with HIV infection has been demonstrated [15,27]. Two of the three included patients with confirmed BoDV-1 infection (patients 2 and 3) in our study were immunosuppressed when blood samples were taken. In all three patients, significantly elevated SFU counts for N protein-derived peptides were found in comparison to the controls. Thus, the BoDV-1 ELISpot appears to be sensitive, even under immunosuppression.

As a BoDV-1-negative validation cohort for test specificity, PBMC samples of patients after solid-organ transplantation with negative BoDV-1 serology were used. As no PBMC samples of patients after solid-organ transplantation with confirmed BoDV-1 infection were available, a validation of the sensitivity of the test in this cohort was currently not possible. The cohort represented patients with complex and variable immune histories after kidney or combined kidney/pancreas transplantation for whom the evaluation of the test performance is of certain interest, as BoDV-1 infections might be a rare differential diagnosis of post-transplantation encephalopathy/encephalitis in endemic regions. As test specificity for this cohort was limited to 89% for stimulation with the N-peptide pool, the BoDV-1 ELISpot results (as well as serological results based on recombinant proteins) have to be interpreted with caution for patients with this characteristic. Limited specificity within this cohort, however, was not caused by a shifted, but rather by a skewed distribution of SFU counts with three outliers. This is reflected by the similar signal-to-noise-ratio (SNR) for healthy blood donors (SNR 10; calculated as ratio of the mean SFU upon stimulation with N peptides for patients with BoDV-1 infection and the mean SFU of healthy blood donors) and for transplant patients (SNR 11). One speculative cause for the reduced specificity of the N-peptide-based BoDV-1 ELISpot in the cohort of patients with solid-organ transplantation might be autoreactivity against expressed endogenous Borna-like elements N (EBLN). EBLN are homologous sequences of the BoDV-1 N protein in the human genome and in the genome of other species [28]. The tissue-dependent transcription of some of these genes as well as the translation of at least the ORF-encoding human EBLN hsEBLN-2 into a functional mitochondrial protein with certain functions was demonstrated [29,30].

An obvious limitation of this pilot study was the small number of included patients suffering from BoDV-1 infections. This is due to the fact that BoDV-1 infections are extremely rare and frozen PBMC samples are not readily available for most BoDV-1 infected patients diagnosed in the past. In addition to the three patients diagnosed at our own institute, we were able to test PBMC samples from four further patients that were kindly provided by an external center. Unfortunately, storage of these samples resulted in an insufficient count of viable cells. For another sample, the positive control did not meet the inclusion criteria. The limited number of viable PBMC also explains why the samples of patients suffering from BoDV-1 infection could not be tested in replicates. Thus, further evaluation of the test is necessary, whenever a PBMC sample of a patient with BoDV-1 infection is available. As all of our tested PBMC samples were taken in the second to third week after hospitalization, early samples after the onset of symptoms should be included in order to address the question of whether the BoDV-1 ELISpot may contribute to a rapid diagnosis of BoDV-1 infections and consecutive early treatment of affected patients. In conclusion, this pilot study provides a proof-of-concept that cellular immune responses in human patients suffering from severe BoDV-1 infection can be detected by a peptide-based BoDV-1 ELISpot. The performance of this test, however, needs to be further validated in patients with BoDV-1 encephalitis.

## Figures and Tables

**Table 1:** Diagnostic tests confirming BoDV-1 infection in the three patients with positive BoDV-1 ELISpot.

	CSF: BoDV-1 RT-qPCR [Copies/mL]	Serum: iIFA [Titer]	Post-Mortem Tissue (Homogenate in Sodium Chloride): BoDV-1 RT-qPCR [Copies/mL]
Patient 1	1 dpa: 1850 4 dpa: not detected 5 dpa: 870 8 dpa: 350	8 dpa: negative (<20) 13 dpa: 320	Frontal cortex: >10^8^ Optic nerve: >10^8^
Patient 2	0 dpa: 520 5 dpa: positive <300 12 dpa: positive <300	0 dpa: negative (<20) 2 dpa: negative (<20) 5 dpa: 80 13 dpa: 320	Not available
Patient 3	17 dpa: positive <300	18 dpa: 40	Frontal cortex: >10^8^ Olfactory bulb: >10^8^ Temporal cortex: >10^8^ Mesencephalon: >10^8^ Pons: >10^8^ Cerebellum: 8.6 × 10^7^

dpa: days post (hospital) admission.

**Table 2:** Comparison of BoDV-1 ELISpot and BoDV-1 ELISA.

	BoDV-1 ELISpot [SFU/250,000 PBMC]	Test Date ELISpot [Days after Hospitalization]	BoDV-1 ELISA [S/CO]	Test Date ELISA [Days after Hospitalization]
Patient 1	N: 20 X: 14 P: 0	13	anti-N-IgG: 0.04 anti-X-IgG: 6.97 anti-P-IgG: 1.13	13
Patient 2	N: 13 X: −1 P: 0	12	anti-N-IgG: 4.66 anti-X-IgG: 7.50 anti-P-IgG: 7.59	12
Patient 3	N: 12 X: 5 P: 9	21	anti-N-IgG: 0.50 anti-X-IgG: 0.64 anti-P-IgG: 1.55	17

SFU: spot-forming units. S/CO: sample to cut-off ratio. For BoDV-1 ELISpot SFU counts, counts of the matched negative controls were subtracted.

## Data Availability

The data presented in this study are available on request from the corresponding author.

## Supplementary Materials

The following supporting information can be downloaded at: https://www.mdpi.com/article/10.3390/v15010194/s1, Figure S1: Flowchart for Borna disease virus 1 (BoDV-1)-specific interferon (IFN)-γ ELISpot assay; Figure S2: BoDV-1 ELISpot pictures; Table S1: Peptide sequences for BoDV-1 ELISpot); Table S2: Excluded samples of patients with BoDV-1 infection; Table S3: Immune history of patients after solid-organ transplantation with focus on BoDV-1 ELISpot outliers.
