# Supplementary material for: IFN-γ-Based ELISpot as a New Tool to Detect Human Infections with Borna Disease Virus 1 (BoDV-1): A Pilot Study
Source: Viruses. 2023 Jan 10;15(1):194. doi: 10.3390/v15010194 (PMC9864614; doi:10.3390/v15010194)
Supplement: Supplementary file 1 [file viruses-15-00194-s001.zip › viruses-2088748-supplementary/viruses-2088748_supplement_Figure S1.pptx]

## Slide 1
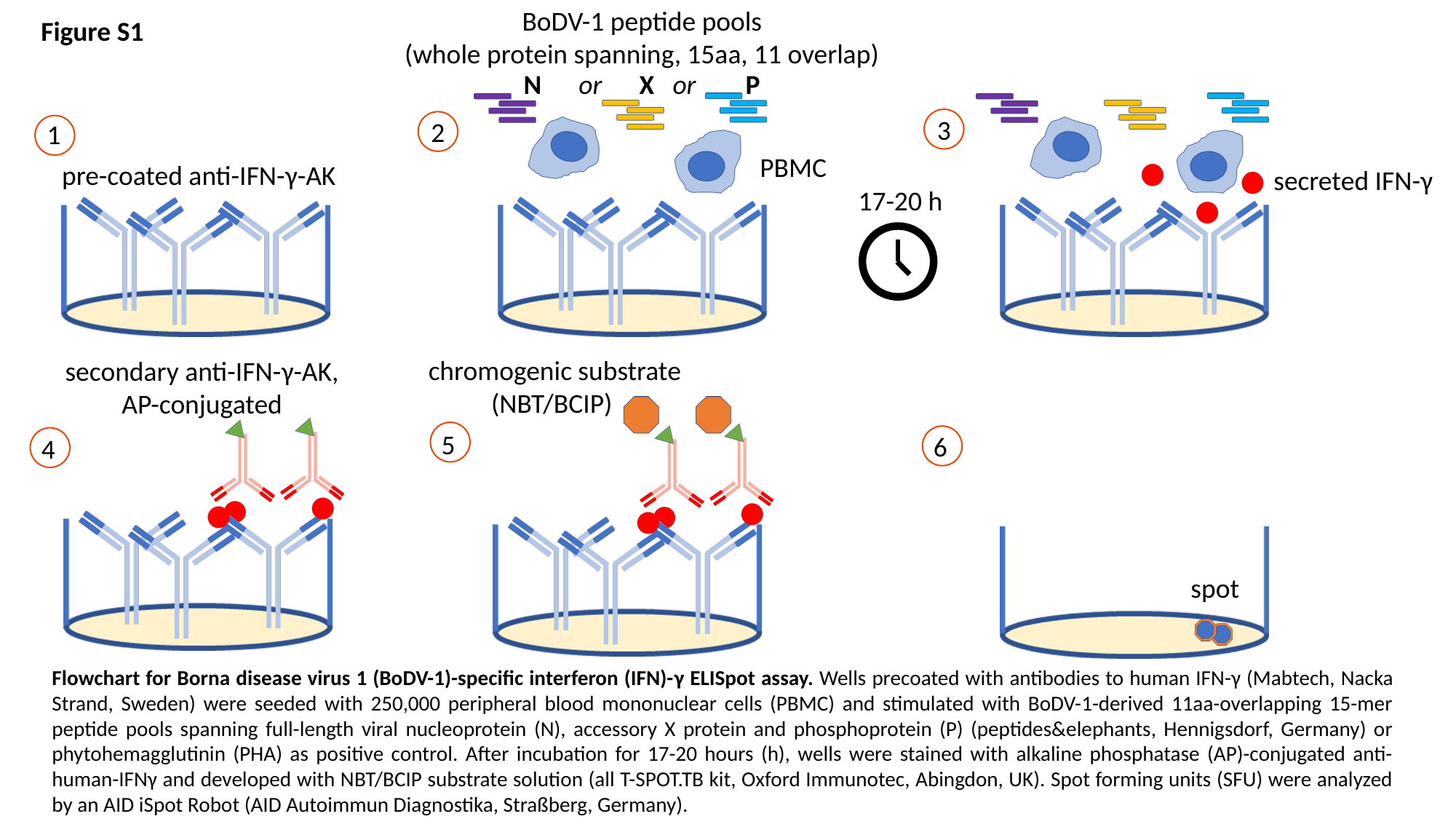

BoDV-1 peptide pools
(whole protein spanning, 15aa, 11 overlap)
Figure S1
N or X or P
3
2
1
PBMC
pre-coated anti-IFN-γ-AK
secreted IFN-γ
17-20 h
chromogenic substrate
(NBT/BCIP)
secondary anti-IFN-γ-AK, AP-conjugated
5
6
4
spot
Flowchart for Borna disease virus 1 (BoDV-1)-specific interferon (IFN)-γ ELISpot assay. Wells precoated with antibodies to human IFN-γ (Mabtech, Nacka Strand, Sweden) were seeded with 250,000 peripheral blood mononuclear cells (PBMC) and stimulated with BoDV-1-derived 11aa-overlapping 15-mer peptide pools spanning full-length viral nucleoprotein (N), accessory X protein and phosphoprotein (P) (peptides&elephants, Hennigsdorf, Germany) or phytohemagglutinin (PHA) as positive control. After incubation for 17-20 hours (h), wells were stained with alkaline phosphatase (AP)-conjugated anti-human-IFNγ and developed with NBT/BCIP substrate solution (all T-SPOT.TB kit, Oxford Immunotec, Abingdon, UK). Spot forming units (SFU) were analyzed by an AID iSpot Robot (AID Autoimmun Diagnostika, Straßberg, Germany).
