# Supplementary material for: IFN-γ-Based ELISpot as a New Tool to Detect Human Infections with Borna Disease Virus 1 (BoDV-1): A Pilot Study
Source: Viruses. 2023 Jan 10;15(1):194. doi: 10.3390/v15010194 (PMC9864614; doi:10.3390/v15010194)
Supplement: Supplementary file 1 [file viruses-15-00194-s001.zip › viruses-2088748-supplementary/viruses-2088748_supplement_Figure S2.pdf]

patients with BoDV-1 infection	negative control	BoDV-1 N peptide pool	BoDV-1 X peptide pool	BoDV-1 P peptide pool	positive control (PHA)
patient 1	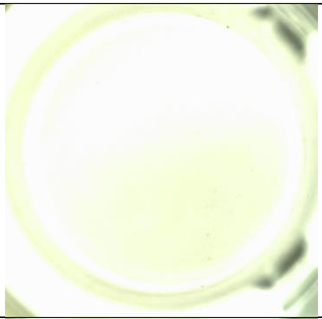	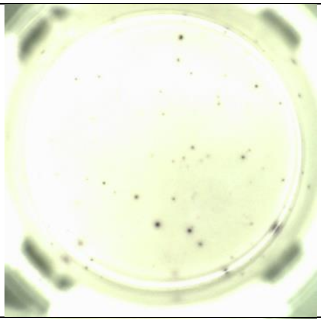	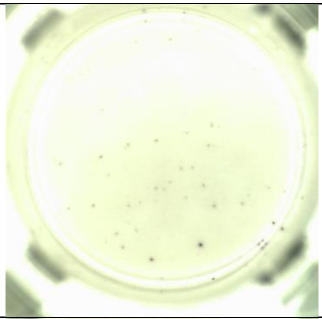	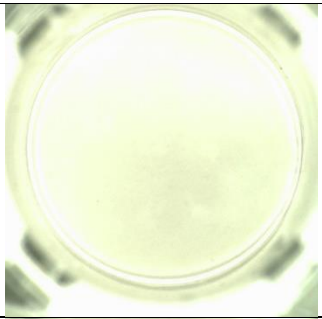	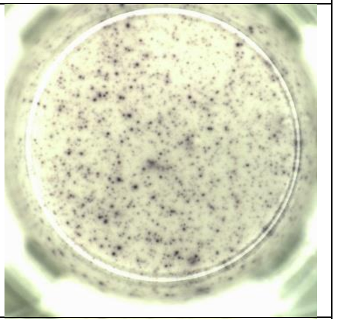
patient 2	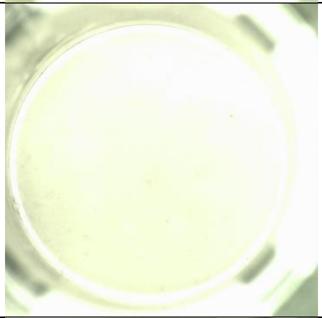	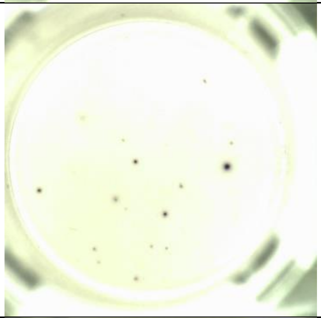	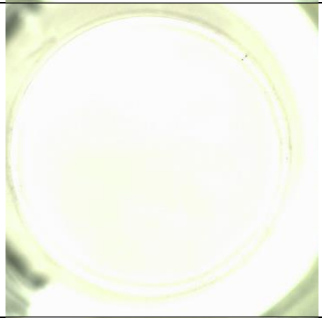	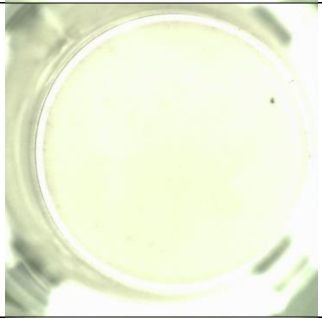	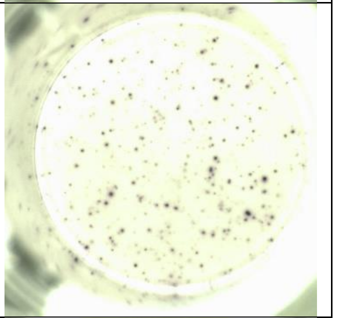
patient 3	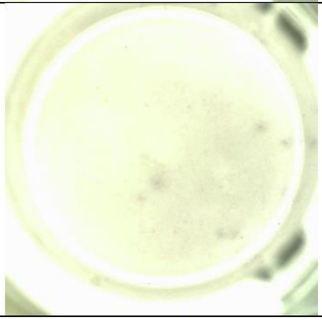	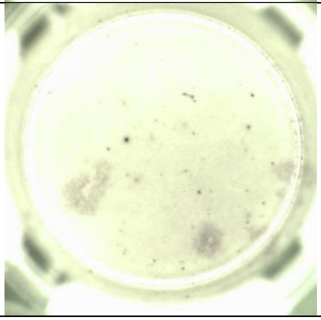	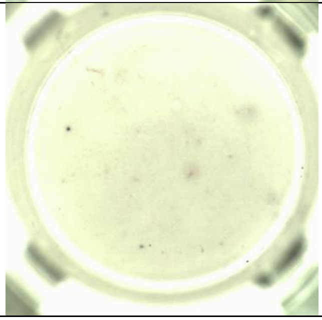	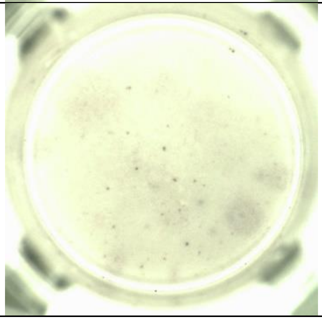	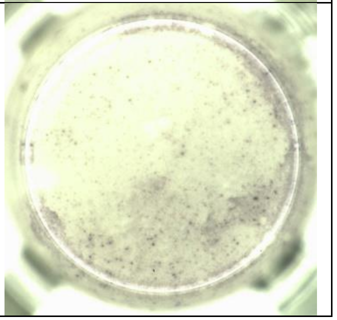

Figure S2. BoDV-1 ELISpot pictures. Unedited pictures as they were collected by an AID iSpot Robot (settings: relative spot intensity ≥ 25, spot size ≥ 100 [0.01 mm^2], gradient ≥ 10) show peripheral blood mononuclear cell (PBMC) samples of three patients with confirmed BoDV-1 infection

without stimulation (negative control), under stimulation with BoDV-1-protein-derived peptides (N, X and P protein) or stimulated with phytohemagglutinin (PHA; positive control). Per well, 250,000 PBMC were seeded. Patient 2 and 3 were under immunosuppressive treatment at the time of PBMC sampling.
