# Supplementary material for: IFN-γ-Based ELISpot as a New Tool to Detect Human Infections with Borna Disease Virus 1 (BoDV-1): A Pilot Study
Source: Viruses. 2023 Jan 10;15(1):194. doi: 10.3390/v15010194 (PMC9864614; doi:10.3390/v15010194)
Supplement: Supplementary file 1 [file viruses-15-00194-s001.zip › viruses-2088748-supplementary/viruses-2088748_supplement_Table S1.pdf]

Table S1. Peptide sequences for BoDV-1 ELISpot.

BoDV-1 protein	peptide sequence (15-mer, 11aa overlap)
BoDV-1 N protein (p40): N → C	MPPKRRLVDDADAME<br>RRLVDDADAMEDQDL<br>DDADAMEDQDLYEPP<br>AMEDQDLYEPPASLP<br>QDLYEPPASLPKLPG<br>EPPASLPKLPGKFLQ<br>SLPKLPGKFLQYTVG<br>LPGKFLQYTVGGSDP<br>FLQYTVGGSDPHPGI<br>TVGGSDPHPGIGHEK<br>SDPHPGIGHEKDIRQ<br>PGIGHEKDIRQNAVA<br>HEKDIRQNAVALLDQ<br>IRQNAVALLDQSRRD<br>AVALLDQSRRDMFHT<br>LDQSRRDMFHTVTPS<br>RRDMFHTVTPSLVFL<br>FHTVTPSLVFLCLLI<br>TPSLVFLCLLIPGLH<br>VFLCLLIPGLHAAFV<br>LLIPGLHAAFVHGGV<br>GLHAAFVHGGVPRES<br>AFVHGGVPRESYLST<br>GGVPRESYLSTPVTR<br>RESYLSTPVTRGEQT<br>LSTPVTRGEQTVVKT<br>VTRGEQTVVKTAKFY<br>EQTVVKTAKFYGEKT<br>VKTAKFYGEKTTQRD<br>KFYGEKTTQRDLTEL<br>EKTTQRDLTELEISS<br>QRDLTELEISSIFSH<br>TELEISSIFSHCCSL<br>ISSIFSHCCSLLIGV<br>FSHCCSLLIGVVIGS<br>CSLLIGVVIGSSSKI<br>IGVVIGSSSKIKAGA<br>IGSSSKIKAGAEQIK<br>SKIKAGAEQIKKRFK<br>AGAEQIKKRFKTMMA<br>QIKKRFKTMMAALNR<br>RFKTMMAALNRPSHG<br>MMAALNRPSHGETAT<br>LNRPSHGETATLLQM<br>SHGETATLLQMFNPH<br>TATLLQMFNPHEAID<br>LQMFNPHEAIDWING<br>NPHEAIDWINGQPWV

	AIDWINGQPWVGSFV<br>INGQPWVGSFVLSLL<br>PWVGSFVLSLLTTDF<br>SFVLSLLTTDFESPG<br>SLLTTDFESPGKEFM<br>TDFESPGKEFMDQIK<br>SPGKEFMDQIKLVAS<br>EFMDQIKLVASYAQM<br>QIKLVASYAQMTTYT<br>VASYAQMTTYTTIKE<br>AQMTTYTTIKEYLAE<br>TYTTIKEYLAECMDA<br>IKEYLAECMDATLTI<br>LAECMDATLTIPVVA<br>MDATLTIPVVAYEIR<br>LTIPVVAYEIRDFLE<br>VVAYEIRDFLEVSAK<br>EIRDFLEVSAKLKED<br>FLEVSAKLKEDHADL<br>SAKLKEDHADLFPFL<br>KEDHADLFPFLGAIR<br>ADLFPFLGAIRHPDA<br>PFLGAIRHPDAIKLA<br>AIRHPDAIKLAPRSF<br>PDAIKLAPRSFPNLA<br>KLAPRSFPNLASAAF<br>RSFPNLASAAFYWSK<br>NLASAAFYWSKKENP<br>AAFYWSKKENPTMAG<br>WSKKENPTMAGYRAS<br>ENPTMAGYRASTIQP<br>MAGYRASTIQPGASV<br>RASTIQPGASVKETQ<br>IQPGASVKETQLARY<br>ASVKETQLARYRRRE<br>ETQLARYRRREISRG<br>ARYRRREISRGEDGA<br>RREISRGEDGAELSG<br>SRGEDGAELSGEISA<br>DGAELSGEISAIMKM<br>LSGEISAIMKMIGVT<br>EISAIMKMIGVTGLN
BoDV-1 X protein (p10): N → C	MSSDLRLTLLELVRR<br>LRLTLLELVRRLNGN<br>LLELVRRLNGNATIE<br>VRRLNGNATIESGRL<br>NGNATIESGRLPGGR<br>TIESGRLPGGRRRSP<br>GRLPGGRRRSPDTTT<br>GGRRRSPDTTTGTTG<br>RSPDTTTGTTGVTKT<br>TTTGTTGVTKTTEGP<br>TTGVTKTTEGPKECI

	TKTTEGPKECIDPTS<br>EGPKECIDPTSRPAP<br>ECIDPTSRPAPEGPQ<br>PTSRPAPEGPQEEPL<br>PAPEGPQEEPLHDLR<br>GPQEEPLHDLRPRPA<br>EPLHDLRPRPANRKG<br>DLRPRPANRKGAAVE
BoDV-1 P protein (p24): N → C	MATRPSSLVDSLEDE<br>PSSLVDSLEDEEDPQ<br>VDSLEDEEDPQTLRR<br>EDEEDPQTLRRERPG<br>DPQTLRRERPGSPRP<br>LRRERPGSPRPRKVP<br>RPGSPRPRKVPRNAL<br>PRPRKVPRNALTQPV<br>KVPRNALTQPVDQLL<br>NALTQPVDQLLKDLR<br>QPVDQLLKDLRKNPS<br>QLLKDLRKNPSMISD<br>DLRKNPSMISDPDQR<br>NPSMISDPDQRTGRE<br>ISDPDQRTGREQLSN<br>DQRTGREQLSNDELI<br>GREQLSNDELIKKLV<br>LSNDELIKKLVTELA<br>ELIKKLVTELAENSM<br>KLVTELAENSMIEAE<br>ELAENSMIEAEEVRG<br>NSMIEAEEVRGTLGD<br>EAEEVRGTLGDISAR<br>VRGTLGDISARIEAG<br>LGDISARIEAGFESL<br>SARIEAGFESLSALQ<br>EAGFESLSALQVETI<br>ESLSALQVETIQTAQ<br>ALQVETIQTAQRCDH<br>ETIQTAQRCDHSDSI<br>TAQRCDHSDSIRILG<br>CDHSDSIRILGENIK<br>DSIRILGENIKILDR<br>ILGENIKILDRSMKT<br>NIKILDRSMKTMMET<br>LDRSMKTMMETMKLM<br>MKTMMETMKLMMEKV<br>METMKLMMEKVDLLY<br>KLMMEKVDLLYASTA<br>EKVDLLYASTAVGTS<br>LLYASTAVGTSAPML<br>STAVGTSAPMLPSHP<br>GTSAPMLPSHPAPPR<br>PMLPSHPAPPRIYPQ<br>SHPAPPRIYPQLPSA

	PPRIYPQLPSAPTTD<br>YPQLPSAPTTDEWDI<br>QLPSAPTTDEWDIIP
