# Supplementary material for: IFN-γ-Based ELISpot as a New Tool to Detect Human Infections with Borna Disease Virus 1 (BoDV-1): A Pilot Study
Source: Viruses. 2023 Jan 10;15(1):194. doi: 10.3390/v15010194 (PMC9864614; doi:10.3390/v15010194)
Supplement: Supplementary file 1 [file viruses-15-00194-s001.zip › viruses-2088748-supplementary/viruses-2088748_supplement_Table S2.pdf]

Table S2. Excluded samples of patients with BoDV-1 infection. Thawed PBMC samples of patients with BoDV-1 infection (patients 4-7) that did not meet test requirements (viable cell count not sufficient for seeding 250,000 viable PBMC per well) or test criteria (≥20 SFU/250,000 PBMC in positive control). These samples were subsequently excluded from analysis.

patients with BoDV-1 infection	seeded viable PBMC per well	SFU under stimulation with BoDV-1 N peptides *	SFU in positive control (PHA) *
patient 4	16,000	0 SFU/16,000 PBMC	-1 SFU/16,000 PBMC
patient 5	100,000	0 SFU/100,000 PBMC	0 SFU/100,000 PBMC
patient 6	100,000	0 SFU/100,000 PBMC	21 SFU/100,000 PBMC
patient 7	250,000	0 SFU/250,000 PBMC	8 SFU/250,000 PBMC

* Spot forming units (SFU) of peripheral blood mononuclear cells (PBMC) upon stimulation with BoDV-1 N peptides and phytohemagglutinin (PHA; positive control) were corrected for the SFU count of the respective negative control.
