# Supplementary material for: IFN-γ-Based ELISpot as a New Tool to Detect Human Infections with Borna Disease Virus 1 (BoDV-1): A Pilot Study
Source: Viruses. 2023 Jan 10;15(1):194. doi: 10.3390/v15010194 (PMC9864614; doi:10.3390/v15010194)
Supplement: Supplementary file 1 [file viruses-15-00194-s001.zip › viruses-2088748-supplementary/viruses-2088748_supplement_Table S3.pdf]

Table S3. Immune history of patients after solid-organ transplantation with focus on BoDV-1 ELISpot outliers (upon stimulation with BoDV-1 N peptides).

patients after solid-organ transplantation	solid-organ transplantation	current immuno-suppressive medication	induction at time of transplantation	AB0-incompa-tibility	time since transplantation [years]	history of organ rejection	basic medical condition leading to nephropathy
outlier 1 (BoDV-1 ELISpot N)	kidney	MMF (360 mg/d)<br><br>tacrolimus	no	no	9	unknown	hypertensive nephropathy
outlier 2 (BoDV-1 ELISpot N)	kidney	MMF (500 mg/d)<br><br>everolimus	no	no	12	unknown	focal segmental glomerulosclerosis
outlier 3 (BoDV-1 ELISpot N)	kidney	MMF (1080 mg/d)<br><br>tacrolimus	basiliximab	no	5	no	unknown
cohort (n = 27)	kidney: 93%<br><br>kidney + pancreas: 7%	MMF: 85% (dosage 360-2000 mg/d)<br><br>tacrolimus: 89%<br><br>glucocorticoids: 33%<br><br>others: 7%	yes: 33%<br><br>no: 67%	yes: 15%<br><br>no: 85%	median: 7<br><br>range: 1-23	yes: 19%<br><br>no: 30%<br><br>unknown: 52%	

MMF: mycophenolate mofetil.
